# Apneic response to fentanyl in adult rats: Role of laryngeal afferents

**DOI:** 10.14814/phy2.15965

**Published:** 2024-03-05

**Authors:** Jianguo Zhuang, Xiuping Gao, Shan Shi, Fadi Xu

**Affiliations:** ^1^ Department of Physiology Lovelace Biomedical Research Institute Albuquerque New Mexico USA

**Keywords:** bradycardia, central apnea, hypertension, laryngeal C‐ and Aδ‐fibers, superior laryngeal nerve

## Abstract

Intravenous (systemic) bolus injection of fentanyl (FNT) reportedly induces an immediate vagal‐mediated apnea; however, the precise origin of vagal afferents responsible for this apnea remains unknown. We tested whether intralaryngeal (local) application of FNT would also trigger an apnea and whether the apneic response to both local and systemic administration of FNT was laryngeal afferent‐mediated. Cardiorespiratory responses to FNT were recorded in anesthetized male adult rats with and without bilateral sectioning of the superior laryngeal nerve (SLNx) or peri‐SLN capsaicin treatment (SLNcap) to block local C‐fiber signal conduction. Opioid mu‐receptor (MOR)‐immunoreactivity was detected in laryngeal C‐ and myelinated neurons. We found that local and systemic administration of FNT elicited an immediate apnea. SLNx, rather than SLNcap, abolished the apneic response to local FNT application though MORs were abundantly expressed in both laryngeal C‐ and myelinated neurons. Importantly, SLNx failed to affect the apneic response to systemic FNT administration. These results lead to the conclusion that laryngeal afferents' MORs are responsible for the apneic response to local, but not systemic, administration of FNT.

## 1 INTRODUCTION

The United States has experienced a troubling increase in overdose opioid‐induced deaths, rising from 75,673 in 2020 to 80,816 in 2021, representing a 15% increase (source: CDC Press Release, 2022). The primary cause of these deaths is the rapid intravenous injection of a lethal dose of fentanyl (FNT) (Comer & Cahill, 2019; Denton et al., 2008; Mercado‐Crespo et al., 2014). The death occurs within a few minutes in both illicit users and clinical patients for managing acute pain (Peng & Sandler, 1999; Richardson & Egan, 2005), especially cancer‐related breakthrough pain (Barnett, 2001; Prommer, 2009). We have reported that intravenous bolus (IVb) injection of FNT is capable of stimulating vagal C‐fibers to produce an immediate apnea with the apneic duration being dose‐dependent in anesthetized rats (Zhuang et al., 2012, 2022). For example, FNT at 25 μg kg^−1^ induced a prolonged apnea lasting 30 s. Because vagal C‐fibers innervate a variety of organs, including the heart, airways, lungs, and abdominal viscera, the precise origin of the vagal C‐fibers responsible for the apneic response to IVb injection of FNT has not been determined.

Among vagal afferents, the sensory nerve endings in the larynx play a pivotal role in the control of respiratory rhythm and upper airway resistance. However, limited information is available regarding whether FNT can directly stimulate laryngeal afferents to induce apnea and thereby contribute to IVb injection‐induced apnea. The superior laryngeal nerve (SLN) primarily provides sensory innervation of the larynx above the vocal cords and is part of the vagus nerve (Hishida et al., 1997; Yoshida et al., 2000). Cell bodies of SLN afferents are located in nodose/jugular ganglia, and their central afferents synapse in the ipsilateral nucleus tractus solitarius, which further project to pontomedullary respiratory‐related nuclei (Hishida et al., 1997; Yoshida et al., 2000). For example, the projections to the nucleus ambiguus contain the preganglionic cardiac vagal cell bodies and the recurrent laryngeal nerve motoneurons (Reix et al., 2007). There are several lines of evidence supporting the involvement of the SLN in FNT‐induced apnea. First, chemical stimulation of the larynx reflexively triggers central expiratory apnea, laryngeal constriction, cough and swallowing associated with bradycardia and hypertension (Abu‐Shaweesh et al., 2001; Lee et al., 1977). In the SLN, C‐ and Aδ‐fibers, especially C‐fibers, mediate the chemoreflex in various animals (Gao et al., 2017; Liu et al., 2013; Mutoh et al., 2000; Wei et al., 2020). Second, endogenous opioids have been reported to modulate the apneic and vocal closure responses to the activation of laryngeal afferents (Jimenez‐Vargas & Carreira‐Monteiro, 1986; Mutolo et al., 1995). Third, IV infusion of FNT or morphine induces apnea and upper airway obstruction in humans and rats (Khalil et al., 2009; Willette et al., 1982). Forth, mu‐opioid receptors (MORs) are expressed in vagal sensory neurons, including C‐neurons (Li et al., 1996; Zhuang et al., 2012) and FNT stimulates these neurons in vitro (Zhuang et al., 2022). Based on these results, we hypothesized that intralaryngeal application of FNT would induce apnea and that both apneic responses to intralaryngeal application and IVb injection of FNT were mediated by the SLN, especially laryngeal C‐fibers.

To test this hypothesis, we recorded cardiorespiratory responses to intralaryngeal application and IVb injection of FNT in anesthetized rats with or without bilateral sectioning of the SLN (SLNx) or peri‐SLN capsaicin treatment (SLNcap). Immunofluorescent MOR expression in laryngeal C‐ and myelinated neurons was detected.

## 2 METHODS

### 2.1 Animal use

Pathogen‐free Sprague–Dawley adult male rats (*n* = 54, 250–350 g) were purchased from Charles River Laboratories, Inc. (Wilmington, MA). They were housed in filter top cages in the animal facility of Lovelace Biomedical Research Institute with 12:12 h light/dark cycle and provided with water and food (16% protein rodent diet) ad libitum. The rooms were constantly ventilated, and the temperature was kept at 24–25°C. The animals were quarantined for 1 week before experiments. Experiments were performed during 9:00 and 17:00 h to avoid influence of the circadian rhythm. The experimental protocols were conducted in accordance with the Guide for the Care and Use of Laboratory Animals and approved by the Institutional Animal Care and Use Committee (IACUC), which is accredited by the Association for Assessment and Accreditation of Laboratory Animal Care International, USA. After completion of the experiments, the rats were euthanized with intraperitoneal injection of 3.0 mL of 1:9 diluted Euthasol (Euthanasia solution, Virbac AH, Inc., TX).

### 2.2 General animal preparation

The rats were anesthetized with urethane (1200 mg kg^−1^, ip) with supplemental dose (300 mg kg^−1^, ip) administered, as needed, to completely suppress eye‐blink and limb‐withdrawal reflex throughout the experiment. The rat was placed in a supine position and the trachea (5 mm below the larynx) was opened with a transverse incision to expose the lumen but leave the posterior tracheal wall intact (Gao et al., 2017; Wei et al., 2020). Both the rostral and caudal segments of the trachea were cannulated separately. The caudal tracheal opening was cannulated and connected to a pneumotachograph to record airflow form which minute ventilation (V_E_), tidal volume (V_T_), respiratory frequency (f_R_), and expiratory duration (T_E_) were derived. The pneumotachograph had a linear flow‐pressure relationship in the range of 2–20 mL s^−1^, a flow resistance of 0.046 cmH_2_O mL^−1^ s, and a dead space of 0.2 mL. The rostral tracheal opening was also cannulated as detailed below. The right femoral artery was cannulated for monitoring of arterial blood pressure (ABP) and heart rate (HR). The animal was exposed to 30% O_2_ throughout the experiment to prevent hypoxia. Its core temperature was monitored with a rectal probe and maintained at 36.5–37.5°C by a heating pad and radiant heat.

### 2.3 Intralaryngeal application of FNT

Briefly (Gao et al., 2017; Liu et al., 2015), a laryngeal catheter (PE‐190) with a premade window (~2.5 mm long x 1.2 mm width) was inserted cranially from the rostral tracheal opening and extended out of the mouth bypassing the pharynx and mouth. The window was positioned near the vocal cord where most of the laryngeal mucosal surface was exposed to the perfused solution. The catheter was then fixed at both ends. The solution in the catheter was perfused from the tracheal end to the mouth end of the catheter at a constant flow rate (10 μL s^−1^, for 2 s) (Gao et al., 2017) by using a programmable single‐syringe infusion pump (model SP100i, WPI, Sarasota, FL). Saline or FNT (15 μg kg^−1^ in 20 μL saline) was perfused into the larynx. Perfusion at this flow rate for 10 s did not induce detectable leaking because the same amount of solution volume was collected from the mouth end of the catheter in our studies. After laryngeal delivery of vehicle or FNT, the catheter was flushed with saline twice.

### 2.4 Experimental protocols

*Study Series I* aimed to determine whether local application of FNT into the larynx would induce cardiorespiratory responses, and if so whether the evoked responses were reproducible. Five min after stabilization of baseline cardiorespiratory activities (the same for the following experiments), vehicle was perfused into the larynx twice with a 2.5‐h interval between the two applications in three rats. The effects of intralaryngeal application of FNT (15 μg kg^−1^, the same for the following local FNT administration) on cardiorespiratory responses were subsequently tested in additional five rats. The application was repeated once to determine the reproducibility of the cardiorespiratory responses with the same interval as described above. FNT at 15 μg kg^−1^ was chosen here because in our previous pilot study intralaryngeal application of FNT at 5–10 μg kg^−1^ failed to induce detectable cardiorespiratory responses in four rats.

*Study Series II* was performed to test the dependency of the cardiorespiratory response to intralaryngeal application of FNT on the integrity of the SLN, especially SLN C‐fibers. Since the experiments described in *Study Series I* had shown an irreproducibility of the cardiorespiratory response to intralaryngeal application of FNT. Thus, the same intralaryngeal application of FNT was conducted only once in the following relevant experiments. In particular, intralaryngeal application of FNT was performed in the rats after sham operation (*n* = 9) and SLNx (*n* = 7) or after peri‐SLN vehicle (SLNveh, *n* = 5) and capsaicin treatment (SLNcap, *n* = 4). In these rats, no intralaryngeal application of vehicle was performed because it did not alter baseline cardiorespiratory activity in *Study Series I*.

*Study Series III* was undertaken to morphologically identify the presence of MOR expression in laryngeal sensory neurons in the nodose/jugular ganglia in five rats with the retrograde tracer fluoro‐gold (FG) previously injected into the SLN. After euthanasia and fixation, immunohistochemical approach was employed. Nodose/jugular neurons labeled by FG were defined as laryngeal sensory neurons. The latter co‐labeled by both SP‐immunoreactivity (ir) and MOR‐ir were identified as laryngeal C‐neurons expressing MORs (Gao et al., 2017), while those co‐labeled by both neurofilament (NF)‐ir and MOR‐ir were defined as myelinated neurons expressing MORs (Zhang et al., 2008).

*Study Series IV* was designed to test whether the apneic responses to IVb injection of FNT were reproducible and whether the SLN was involved in this response. FNT (5 μg kg^−1^, 50 μL) was applied twice via IVb injection with a 2.5‐h apart to test the reproducibility of the apneic response to systemic administration of FNT (*n* = 8). As the apneic responses to the first and second injections of FNT were reproducible, the same IVb injection of FNT was also performed before (sham operation control) and after SLNx in additional eight rats.

### 2.5 Chemicals and primary antibodies

All chemicals including capsaicin (Cat# M2028) and FNT (fentanyl citrate salt, Cat# F3886) were purchased from Millipore Sigma, St. Louis, MO. A stock solution of capsaicin (1.0 mg mL^−1^) was prepared in 10% Tween 80, 10% ethanol, and 80% saline and subsequently diluted with normal saline to 0.25 mg mL^−1^ for perineural application or other concentrations as needed. FNT was prepared in saline to a stock solution (0.5 mg mL^−1^) and subsequently diluted as needed. Fluoro‐gold (FG, Cat# Fluoro‐gold, 20 mg) was purchased from Fluorochrome, LLC, Denver, CO. Guinea pig anti‐SP IgG (Cat# GP14110) was purchased from Neuromics, Edina, MN. Rabbit anti‐MOR IgG (Cat# AB5511) and mouse anti‐NF IgG (Cat# N2912) were purchased from Millipore Sigma.

### 2.6 Secondary antibodies

Goat anti‐guinea pig (Cat# A11073) or mouse IgG conjugated with Alexa Fluor 488 (Cat# A11029) and goat anti‐rabbit IgG conjugated with Alexa Fluor 594 (Cat# A11037) were purchased from ThermoFisher Sci, Waltham, MA.

### 2.7 Data acquisition and statistical analysis

Digitized raw data of respiratory air flow and ABP as well as their derived data including V_T_, f_R_, V_E_, T_E_, HR, and mean ABP (MABP) were recorded using a PowerLab/8sp unit (model ML 785, ADInstruments Inc., Colorado Springs, CO) and a computer with LabChart Pro 7 software. T_E_ longer than or equal to three‐fold of the baseline T_E_ was defined as an apnea (Pendlebury et al., 2008). All variables were expressed as absolute values with the exception that T_E_, HR, and MABP responses to FNT were presented as ∆folds or ∆% change from baselines (before FNT injection). Group data were reported as means ± *SD*. Repeated Student's *t*‐test was used to compare the differences of cardiorespiratory activity between: baseline “0” and the evoked response (∆% change from baseline); the responses to the first and second FNT administrations. Group Student's *t*‐test was used to compare the differences in the responses to FNT in the rats without and with SLNx or SLNcap. *p*‐values < 0.05 were considered significant.

### 2.8 Retrograde labeling of laryngeal sensory neurons in the nodose/jugular ganglia

Rats were anesthetized with continuous inhalation of isoflurane (1%–2%) administered via a nose cone connected to a vaporizing machine (SurgiVet, Waukesha, WI). A midline incision (1 ~ 1.5 cm) was made in the neck to expose both sides of the SLN. As reported previously (Gao et al., 2017), a beveled glass micropipette needle (10‐μm tip diameter) was pulled by a DMZ universal puller (Dagon Corp., Minneapolis, MN), sharpened with a micro forge (MF‐900, Narishige, Japan), and connected to a Harvard PHD syringe pump (Harvard Apparatus). The micropipette needle was filled with 4% FG solution (dissolved in normal saline), a retrograde tracer to mark laryngeal neurons within the nodose/jugular ganglia. The tip of the needle was gently inserted into the isolated SLN to microinject 0.5 μL of FG solution at a rate of 0.1 μL/min. The skin incision was sutured following bilateral microinjections. Nine to 12 days later, the animals were euthanized and bilateral nodose ganglia were collected for immunohistochemistry.

### 2.9 SLNcap

SLNcap has been previously used in rats and dogs, in which capsaicin blocks the C‐fiber signal conduction (Liu et al., 2015; Mutoh et al., 2000). Briefly, a segment (~2 mm) of the SLN was bilaterally exposed. A vertical slitting in the diameter line was made in a PE50 catheter with a length equal to 2 mm to make two grooved pieces. The segments of the isolated SLN on both sides were gently positioned into the grooves. The edge of the groove was bedded and covered with petroleum jelly so that the SLN segment within the groove was isolated from neighboring tissues. Capsaicin (0.25 mg mL^−1^, 2–3 μL) or vehicle was carefully injected into the grooves to immerse the SLN segments. The solution in the grooves was removed 20 min later with a subsequent saline washout (twice). The successful blockade of C‐fiber signal conduction was confirmed by the lack of apneic response to intralaryngeal infusion of capsaicin (15 μg mL^−1^, 20 μL).

### 2.10 Immunohistochemistry

As previously reported (Gao et al., 2017; Zhuang et al., 2015), rats were perfused transcardially with 30 mL of saline followed by 150 mL of 4% paraformaldehyde in 0.1 M phosphate buffered saline (PBS, pH 7.4). The bilateral nodose ganglia were removed and post‐fixed for 2 h in the same fixative solution. The tissue blocks were rinsed in PBS, cryoprotected overnight in 30% sucrose in PBS, and then embedded in Tissue‐Tek optimal cutting temperature (OCT) embedding medium (Cat# 4583, Sakura Finetek USA, Inc., CA). The ganglia from each animal were serially sectioned (10 μm thick) using a precision cryostat, and three sections with the largest areas were mounted on a gelatin‐chromium‐coated slide. Slides were dried at room temperature for 1 h and washed in PBS, blocked for 1 h in PBS containing 0.1% Triton X‐100 and 10% goat serum, and then incubated overnight at 4°C with primary antibody mixture. One group of sections was incubated with MOR and SP primary antibody (rabbit anti‐MOR, 1:1000; guinea pig anti‐SP IgG, 1:200) diluted in the blocking solution, and another group of sections was incubated with MOR and NF primary antibody (rabbit anti‐MOR, 1:1000; mouse anti‐NF, 1:100). The immunoreactivities were detected using 1:200 secondary antibodies conjugated with Alexa Fluors and visualized using 10× or 40× objective lenses of an epifluorescent microscope (Axioskop FS 2 plus; Zeiss) equipped with fluorescence filters. Images were captured using a digital camera (Zeiss Axiocam HRm) and Zeiss AxioVision 4.8 software. To analyze the immunoreactivity, one representative section with the most numerous FG‐labeled neurons from each of the bilateral nodose ganglia was analyzed. The cells double‐labeled with SP‐ir + MOR‐ir or NF‐ir + MOR‐ir were defined as vagal C‐ or myelinated neurons expressing MORs, while those triple‐labeled with FG + SP‐ir + MOR‐ir or FG + NF‐ir + MOR‐ir were defined as laryngeal C‐ and myelinated neurons expressing MORs. The percentage of the number of neurons double‐labeled with SP‐ir + MOR‐ir or NF‐ir + MOR‐ir relative to the number of those labeled by SP‐ir (vagal C‐neurons) or NF‐ir (vagal myelinated neurons) was counted. It was the same for the number of neurons triple‐labeled with FG + SP‐ir + MOR‐ir or FG + NF‐ir + MOR‐ir relative to the number of those labeled by FG (laryngeal neurons). Cytosolic FG and SP‐, MOR‐, and NF‐ir in each neuron from individual sections was manually determined.

## 3 RESULTS

### 3.1 Intralaryngeal application of FNT triggers a brief apnea that are irreproducible

**FIGURE 1:**
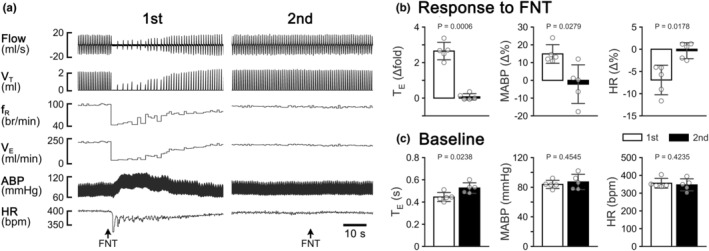
(a) Typical recordings showing that the cardiorespiratory responses to the first and second intralaryngeal application of FNT (15 μg kg^−1^, 2.5 h apart) in an anesthetized rat. The traces from the top to the bottom are air flow, tidal volume (V_T_), respiratory frequency (f_R_), minute ventilation (V_E_), arterial blood pressure (ABP), and heart rate (HR). (b) Group data comparing the cardiorespiratory responses to the first and the second intralaryngeal application of FNT. (c) Baseline cardiorespiratory variables of the first and second intralaryngeal application of FNT. *n* = 5; mean ± *SD*.

Intralaryngeal application of vehicle did not significantly change baseline cardiorespiratory activities, as previously reported (Gao et al., 2017; Wei et al., 2020; Zhuang et al., 2021). However, intralaryngeal application of FNT induced an immediate apnea (lasting for < 1.5 s) associated with hypertension and bradycardia as presented in Figure 1a. In some cases, the evoked apnea was followed by a few T_E_ prolongations. The cardiovascular responses disappeared within 10 min, while V_E_ was recovered approximately 20 min post‐intralaryngeal application of FNT. Surprisingly, the second FNT application applied 2.5 h after the first one did not induce remarkable changes in V_E_, HR, and MABP. Similar to the typical recordings, the corresponding group data showed that the first, but not the second, intralaryngeal application of FNT induced a brief apnea associated with hypertension and bradycardia (Figure 1b). Additionally, the MABP and HR obtained before the first and the second intralaryngeal application of FNT were not significantly different, but the baseline T_E_ appeared to be longer after the first intralaryngeal application of FNT (Figure 1c).

### 3.2 The cardiorespiratory response to intralaryngeal perfusion of FNT depends on the integrity of the SLN

**FIGURE 2:**
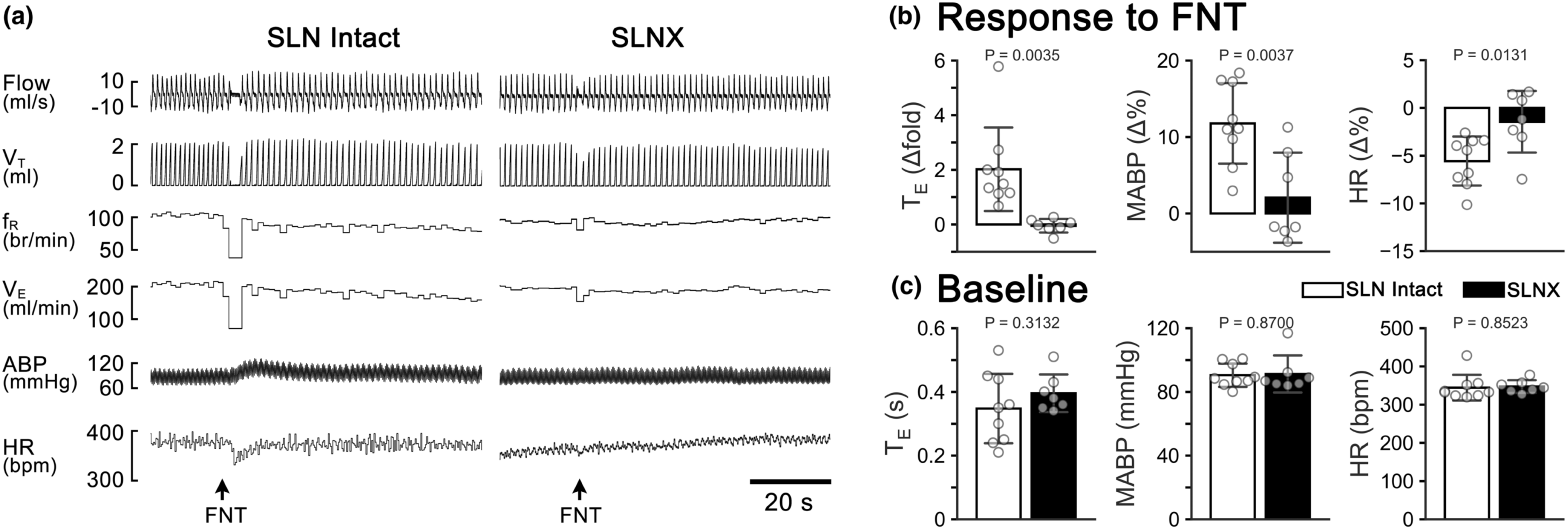
(a) The cardiorespiratory response to intralaryngeal infusion of FNT (15 μg kg^−1^) in two anesthetized rats with the SLN‐intact and SLN‐sectioned (SLNx respectively). The traces from the top to the bottom are air flow, tidal volume (V_T_), respiratory frequency (f_R_), minute ventilation (V_E_), arterial blood pressure (ABP), and heart rate (HR). (b) Group data of the cardiorespiratory responses to intralaryngeal application of FNT in SLN‐intact (*n* = 9) and sectioned rats (*n* = 7). (c) Comparison of baseline cardiorespiratory variables in the intact and SLNx rats. mean ± *SD*.

This study aimed to determine whether the responses to intralaryngeal application of FNT were mediated by the SLN. We compared the cardiorespiratory responses in the rats without and with SLNx and found that SLNx eliminated the cardiorespiratory response to intralaryngeal perfusion of FNT. Typical recordings and the groups data are presented in Figure 2a,b respectively. Moreover, SLNx per se did not cause the significant cardiorespiratory change (Figure 2c).

### 3.3 SLNcap fails to change the cardiorespiratory responses to intralaryngeal application of FNT

**FIGURE 3:**
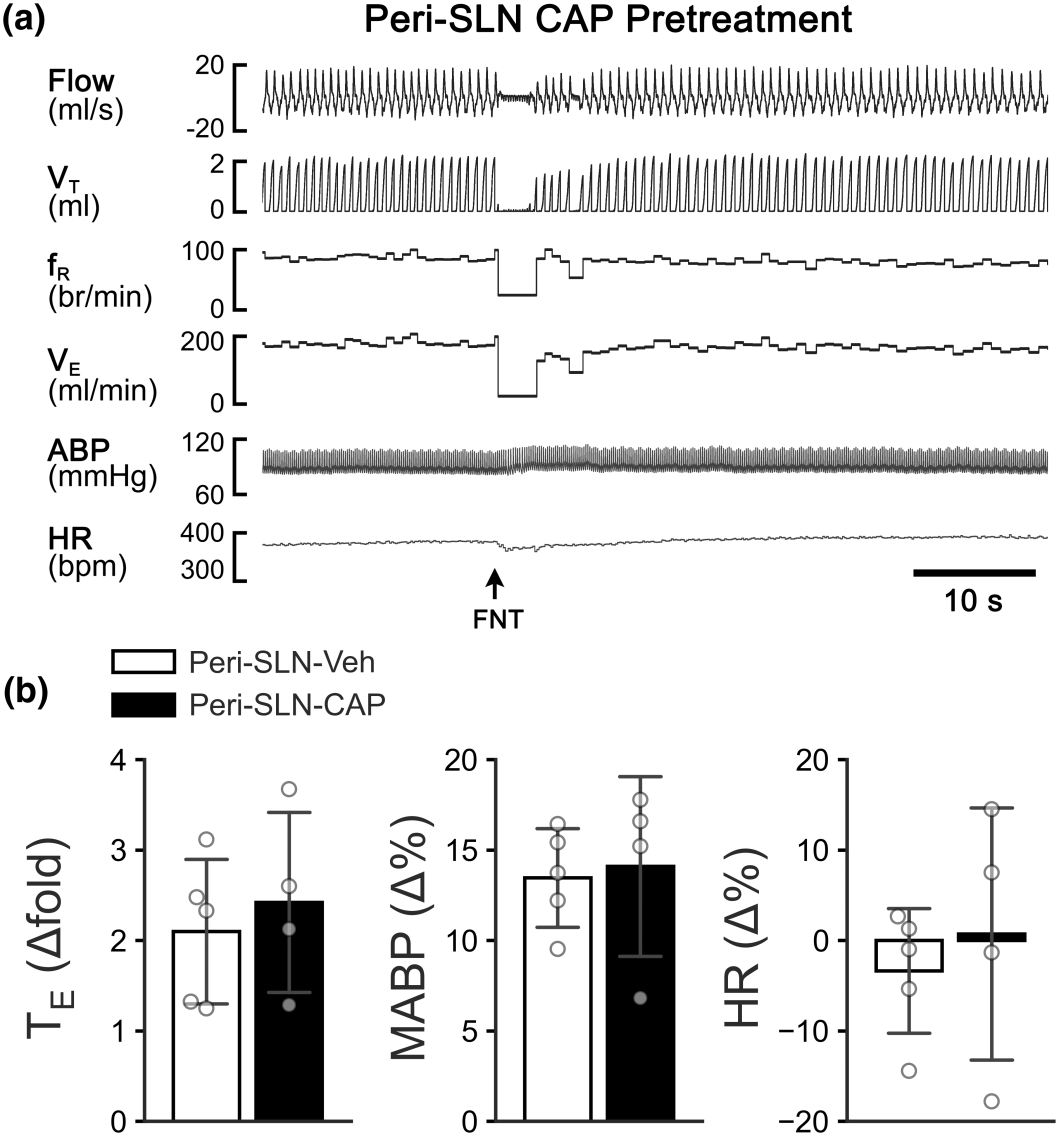
The cardiorespiratory response to intralaryngeal application of FNT (15 μg kg^−1^) in the anesthetized rats treated with SLNveh or SLNcap. (a) Typical recordings showing the responses in a rat with peri‐SLN capsaicin treatment. The traces from the top to the bottom are air flow, tidal volume (V_T_), respiratory frequency (f_R_), minute ventilation (V_E_), arterial blood pressure (ABP), and heart rate (HR). (b) Group data comparing the of cardiorespiratory responses to intralaryngeal infusion of FNT in the SLNveh‐ (*n* = 5) or SLNcap‐treated rats (*n* = 4). Mean ± *SD*.

To verify the role of laryngeal C‐fibers in the cardiorespiratory responses to intralaryngeal application of FNT, the evoked responses were compared between the rats treated with SLNveh and SLNcap. Compared to SLNveh, SLNcap did not significantly alter the cardiorespiratory responses to intralaryngeal application of FNT (Figure 3a,b). It should be noted that neither SLNveh nor SLNcap per se significantly altered baseline cardiorespiratory activities (Table 1).

**TABLE 1:** Effects of SLNcap on baseline cardiorespiratory activities (*n* = 5 for SLNveh and SLNcap respectively; mean ± *SD*).

Variables	Peri‐SLN treatment	
	SLNveh	SLNcap
V_T_ (mL kg^−1^)	8.2 ± 1.3	8.5 ± 1.6
f_R_ (breath min^−1^)	122 ± 16	127 ± 13
V_E_ (mL min^−1^ kg^−1^)	948 ± 22	982 ± 29
T_E_ (s)	0.36 ± 0.13	0.32 ± 0.09
HR (bpm)	349 ± 18	341 ± 27
MABP (mmHg)	81 ± 17	80 ± 20

### 3.4 MORs express in both laryngeal C‐ and myelinated neurons

**FIGURE 4:**
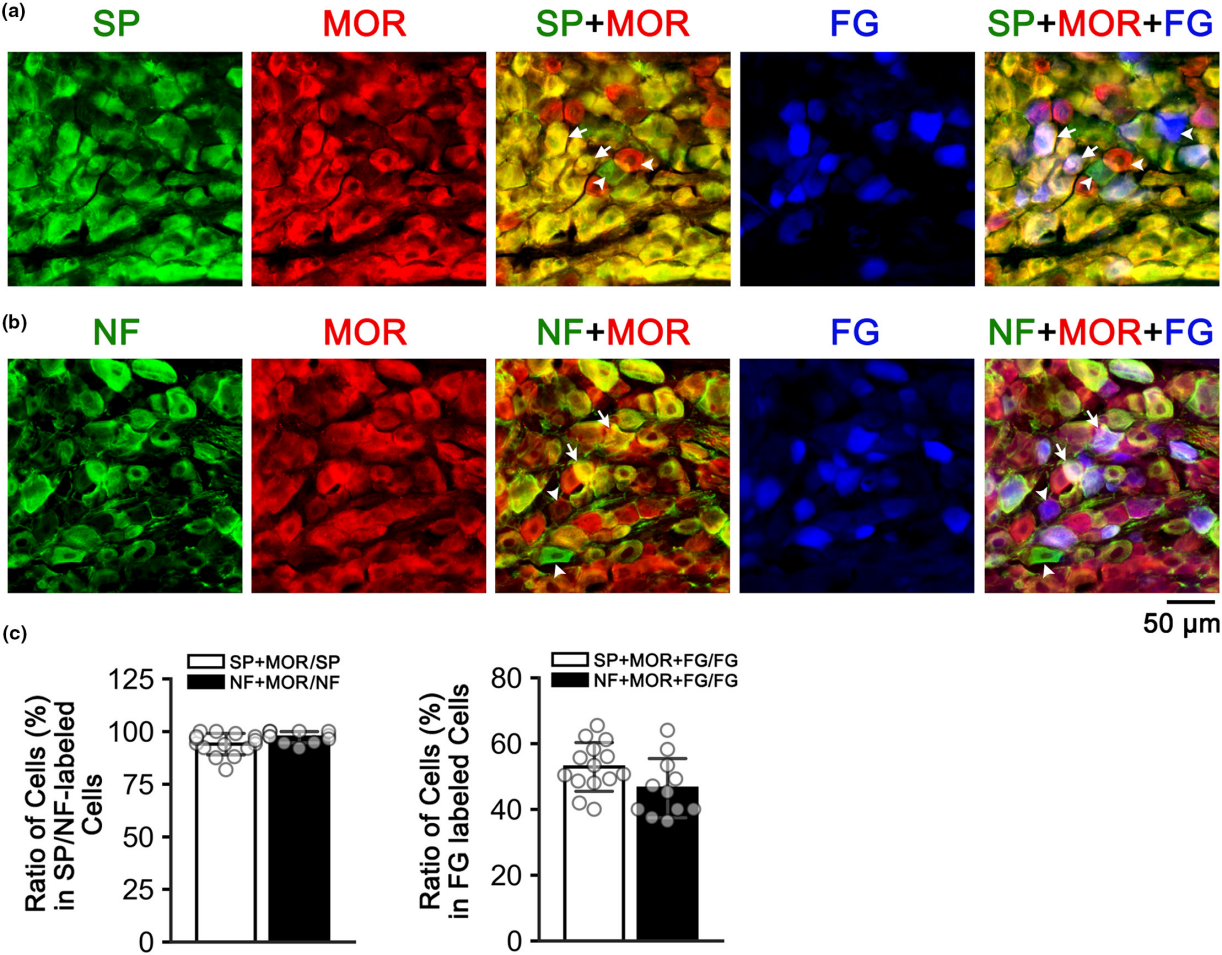
(a) Nodose ganglion neurons labeled by SP‐ir (red), MOR‐ir (green), SP‐ir + MOR‐ir (double‐labeled, yellow), FG (blue), and FG + SP‐ir + MOR‐ir (triple‐labeled, bright purplish). (b): Nodose ganglion neurons labeled by NF‐ir (red), MOR‐ir (green), NF‐ir + MOR‐ir (double‐labeled, yellow), FG (blue), and FG + SP‐ir + MOR‐ir (triple‐labeled, bright purplish). The arrowheads point to single‐labeled neurons, while the arrows point to double‐labeled neurons in SP‐ir + MOR‐ir or NF‐ir + MOR‐ir, or triple‐labeled neurons in FG + SP‐ir + MOR‐ir or FG + NF‐ir + MOR‐ir. (c) The group data (mean ± *SD*) show that the percentage of the number of vagal sensory neurons expressing both SP‐ir + MOR‐ir or NF‐ir + MOR‐ir over the number of those neurons only labeled by SP‐ir or NF‐ir (left panel). In addition, the data also show the percentage of laryngeal neurons triple‐labeled by FG + SP‐ir + MOR‐ir or FG + NF‐ir + MOR‐ir within laryngeal sensory neurons marked by FG (right panel).

The typical images and the corresponding group data are depicted in Figure 4. We found that MOR‐ir was expressed in 94% of vagal C‐neurons and 97% in vagal myelinated neurons. With respect to laryngeal sensory neurons, MOR‐ir was expressed in 53% (C‐) and 46% (myelinated‐) laryngeal neurons. These data indicate that similar to vagal cells, most of laryngeal C‐ and myelinated neurons express MORs.

### 3.5 The apneic response to IVb injection of FNT is not affected by sectioning of the SLN

**FIGURE 5:**
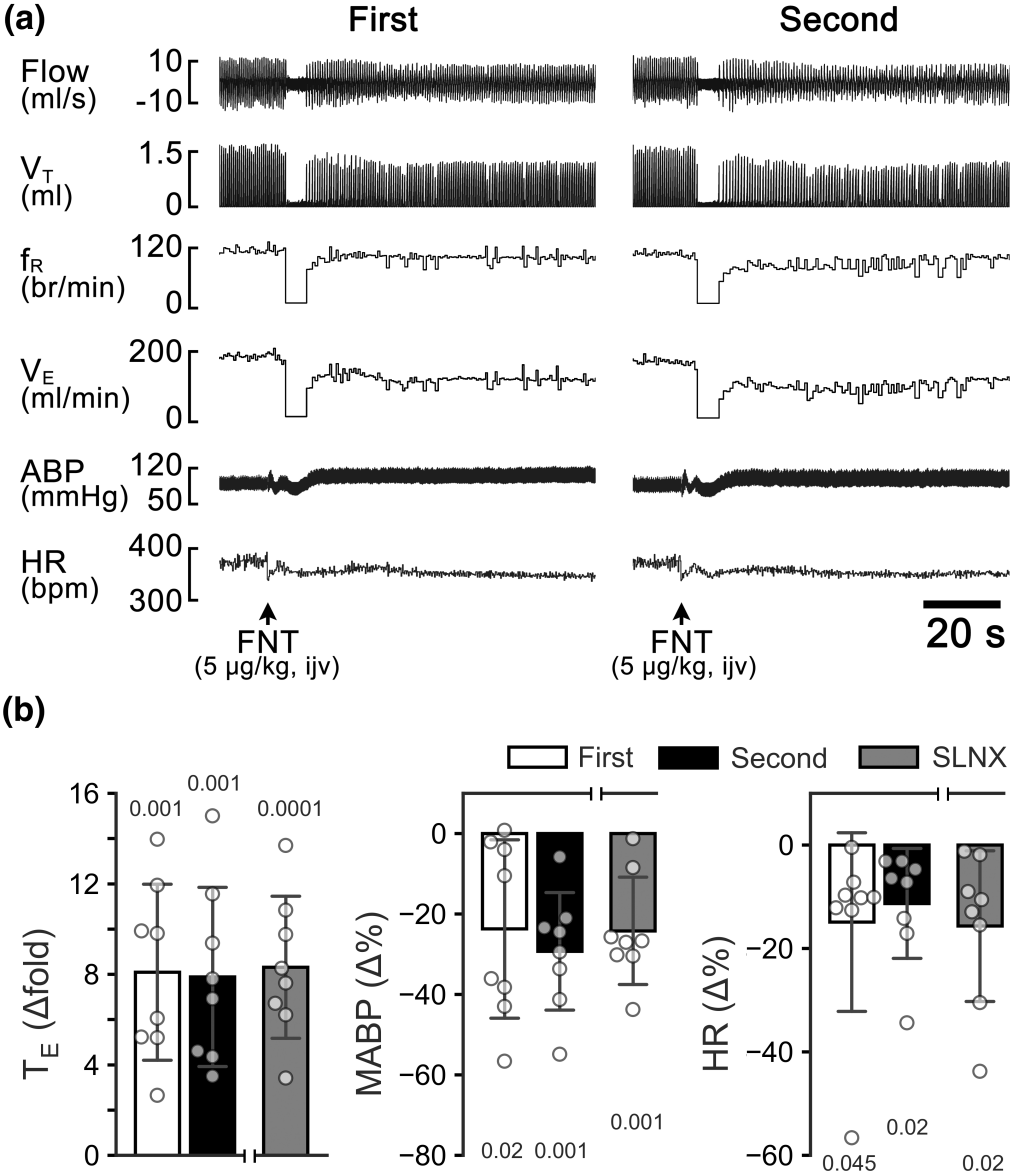
The cardiorespiratory response to IVb injection of FNT (5 μg kg^−1^) in the anesthetized rats. (a) Typical recordings of the cardiorespiratory responses to the first and second IVb injection of FNT in a rat. The traces from the top to the bottom are air flow, tidal volume (V_T_), respiratory frequency (f_R_), minute ventilation (V_E_), arterial blood pressure (ABP), and heart rate (HR). (b) The group data showing the cardiorespiratory responses to the first and second IVb injection of FNT, and to FNT after sectioning of the SLN. *n* = 8 for the reproducibility and *n* = 8 for after SLNx; mean ± *SD*.

The role of the SLN in inducing the apneic response to FNT applied in the larynx raised a fundamental question as to whether the SLN was also important in the apneic response to IVb injection of FNT. To address this issue, the cardiorespiratory responses to IVb injection of FNT were compared in the rats before and after SLNx. As illustrated in Figure 5a,b, FNT administered by IVb injection induced an immediate apnea (lasting > 5.0 s) accompanied with hypotension and bradycardia, which were reproducible. These responses were not significantly affected by SLNx.

## 4 DISCUSSION

The significant findings of our study can be summarized as follow: (1) Intralaryngeal application of FNT (15 μg kg^−1^) resulted in a brief apnea (<1.5 s), accompanied by hypertension and bradycardia. Importantly, these responses disappeared after SLNx, but not SLNcap. (2) MORs were expressed in the majority of laryngeal C‐ and myelinated neurons. (3) Compared to the brief apneic response to local application of FNT, IVb injection of FNT (5 μg kg^−1^) induced a much longer apnea (>5.0 s) associated with hypotension and bradycardia. Notably, these effects were not affected by SLNx.

Previous studies have shown that stimulation of the glottis in dogs (Jimenez Vargas et al., 1987; Jimenez‐Vargas & Carreira‐Monteiro, 1986) and the SLN in cats (Mutolo et al., 1995) induce an immediate apnea, which is greatly attenuated by naloxone administration. These findings suggest a modulatory effect of endogenous opioids on the apneic and vocal closure responses triggered by activation of laryngeal afferents. Additionally, IVb injection of morphine causes immediate excitation of the recurrent laryngeal nerve and augmentation of laryngeal resistance in anesthetized rats (Willette et al., 1982), indicating a stimulatory effect of systemic administration of opioids on laryngeal efferents. While the sensitivity of the SLN to chemicals and its ability to induce immediate apnea, bradycardia, and hypertension have been well‐documented in previous studies (Bohm et al., 2007; Carreau et al., 2011; Forsberg et al., 1988; Gao et al., 2017; Liu et al., 2013; Miller, 1976; Mutoh et al., 2000; Wei et al., 2020), it is still unknown whether opioids applied directly onto the larynx could affect cardiorespiratory activities. Our study introduces a novel concept by demonstrating that the application of FNT into the larynx of anesthetized rats can trigger a brief apnea, bradycardia, and hypertension. Moreover, our finding that laryngeal sensory neurons abundantly express MORs suggests that the stimulation of MORs on laryngeal sensory endings can initiate these cardiorespiratory responses. Consistent with our finding, previous research has indicated that vagal sensory neurons express MORs (Li et al., 1996;Zhuang et al., 2012, 2022) and that MOR agonists stimulate dorsal root ganglionic and vagal afferent neurons (Chen et al., 1988; Shen & Crain, 1990; Zhuang et al., 2022).

Two notable features of the apneic response to intralaryngeal application of FNT stand out, differing from the apnea observed in previous reports. One is that the apneic duration (T_E_ lasting < 1.5 s) induced by FNT (15 μg mL^−1^) in this study is considerably shorter than that (T_E_ lasting >4  s) in response to intralaryngeal application of capsaicin (15 μg mL^−1^) in rats, (Gao et al., 2017; Wei et al., 2020). This divergence may be attributed to the laryngeal chemosensitivity being lower to FNT than capsaicin. Indeed, it has been demonstrated that the excitatory response of vagal C‐neurons to FNT is smaller than their response to capsaicin (Zhuang et al., 2022). Another feature is that the apneic response to intralaryngeal application of FNT is not reproducible, at least 2.5 h after the first application as shown in Figure 1. In sharp contrast, the apneic response to IVb administration of FNT is reproducible (Figure 5), similar to the previous reports (Zhuang et al., 2012, 2022). We propose that this laryngeal desensitization of FNT results from the prolonged internalization of local MORs, as MOR desensitization is known to be correlated to its internalization (Ferguson et al., 1996).

The sensory nerve endings of the SLN consist primarily of unmyelinated C‐fibers and myelinated Aδ‐fibers (Chung & Sant'Ambrogio, 1993; Hishida et al., 1997; Widdicombe & Tatar, 1988). It has been observed in the rats that approximately 49.5% of SLN fibers (comprising 42.5% Aδ‐fibers and 7% Aβ‐fibers) are myelinated fibers, while the remaining 51.5% are unmyelinated C‐fibers (Hishida et al., 1997). These sensory fibers are responsible for two types of laryngeal reflexes by activation of different receptors. One is laryngeal chemoreflex, elicited by substances like capsaicin, acid, and distilled water (Boggs & Bartlett, 1982; Downing & Lee, 1975; Lee et al., 1977; Mutoh et al., 2000; St‐Hilaire et al., 2005). The other type is the laryngeal mechanoreflex, triggered by stimuli, such as air pulse/pressure, air puff, and mucosal vibration (Aviv et al., 1993; Boushey et al., 1974; Murakami & Kirchner, 1972). In addition to SLN C‐fibers, Aδ‐fibers are also responsive to chemical stimulations (Boushey et al., 1974), including acid, bradykinin, and histamine (Hough & Rice, 2011; Kajekar et al., 1999; Kollarik & Undem, 2002). For example, acid stimulates C‐fibers via activation of transient receptor potential vanilloid 1, whereas Aδ‐fibers respond to acid through acid sensing ion channels (Kollarik & Undem, 2002). To investigate whether laryngeal afferents, particularly laryngeal C‐fibers, mediate the apneic response to intralaryngeal application of FNT, we conducted experiments to compare the apneic response before and after intervention of laryngeal afferents. We found that SLNx, but not SLNcap, abolished the intralaryngeal application of FNT‐induced apnea, bradycardia, and hypertension. Moreover, almost all laryngeal sensory neurons labeled by SP‐ir or NF‐ir express MOR‐ir in this study (as shown in Figure 4), suggesting that the majority of laryngeal unmyelinated and myelinated sensory neurons express MORs. The chemosensitivity of Aδ‐fibers and the predominate population of laryngeal Aδ‐fibers in myelinated fibers (Hishida et al., 1997; Sessle, 1973) coupled with the lack of the effect of SLNcap on the response to local application of FNT, suggesting an involvement of laryngeal afferents, presumably laryngeal Aδ‐fibers, in the cardiorespiratory response. A recent study reported that the transient receptor potential cation channel subfamily V member 1, as the capsaicin receptor, was expressed in both C‐ and Aδ‐neurons innervating the bone (Morgan et al., 2019). In agreement, capsaicin is capable of stimulating Aδ‐fibers though the proportion of sensory afferents to be stimulated by capsaicin is significantly greater in C‐fibers than Aδ‐fibers (Kaufman et al., 1982). These reports raise a possibility that perineural capsaicin treatment of the vagus nerve used in this study may not be highly selective to C‐fibers in the SLN. Because cooling of the vagus nerve to 5–7°C and 0°C is able to differentially block C‐ and myelinated‐fiber signal conduction respectively (Roberts et al., 2015; Simera et al., 2016), this method will be employed in future to verify the involvement of SLN myelinated fibers in the apneic response to intralaryngeal application of FNT. Moreover, the exact function of laryngeal C‐fibers expressing MORs remains unknown and warrants investigation.

We recently reported that the apneic duration in response to IVb injection of FNT was dose‐dependent in anesthetized rats (Zhuang et al., 2022). Actually, we observed in a pilot study that FNT administered at >50 μg kg^−1^ led to sudden death within 4–5 min in two anesthetized rats (Zhuang et al, unpublished observation). This observation is consistent with the sudden death induced by IVb injection of overdose FNT in illicit abusers and some patients (Barnett, 2001; Burns et al., 2016; Comer & Cahill, 2019; Denton et al., 2008; Mercado‐Crespo et al., 2014; Peng & Sandler, 1999; Prommer, 2009; Richardson & Egan, 2005). Interestingly, it has been documented that the apneic response to IVb injection of MOR agonists, such as FNT and morphine, is mediated by vagal afferents in anesthetized rats (Willette & Sapru, 1982; Wojciechowski et al., 2007; Zhuang et al., 2012, 2022). Among vagal afferents, the role of laryngeal sensory afferents in the genesis of sudden death has been a subject of specific interest for some time. Laryngeal‐mediated apnea has been implicated as a contributing factor in some cases of sudden infant death syndrome (SIDS) (Duke et al., 2001; Page & Jeffery, 2000; Richardson & Adams, 2005; Thach, 1997). For example, a sibling of one SIDS victim, who exhibited a remarkable prolonged apneic response to stimulation of laryngeal afferents, also succumbed to SIDS (Wennergren et al., 1989). Hypoxemia has been identified as an acute precursor of SIDS (Daley, 2004; Kinney & Thach, 2009), and it can sensitize the SLN‐mediated apnea in infants and neonatal/postnatal animals (Downing & Lee, 1975; Lanier et al., 1983; Wennergren et al., 1989). Severe hypoxia (PaO_2_ < 60 mmHg) has been shown to trigger firings of the SLN, leading to exaggerated glottal abduction (Moraes & Machado, 2015; O'Leary et al., 2004). In some cases, laryngeal stimulation during hypoxia has resulted in significantly prolonged apneas that required resuscitation in piglets (Goding, 1998; Heman‐Ackah, 2005). Given the potential impact of laryngeal‐mediated apnea on the pathogenesis of SIDS and the role of laryngeal afferents' MORs in triggering the apnea in this study, we subsequently investigated if laryngeal afferents were indispensable for the apneic response to IVb injection of FNT in the present study. Surprisingly, we found that SLNx failed to change the apneic response to IVb injection of FNT. As a result, the examination of the influence of laryngeal C‐fibers traveling within the SLN on the IVb injection‐induced apnea became unwarranted. Our results lead us to conclude that laryngeal afferents do not play a significant role in the long‐lasting apneic response to IVb injection of FNT.

In conclusion, our data shed light on the respiratory impacts of FNT when acting on laryngeal afferents. We observed distinct patterns of cardiorespiratory response to FNT administration via intralaryngeal application and IVb injection. Intralaryngeal application of FNT produces a brief apnea associated with hypertension and bradycardia dependent on the integrity of the SLN. The evoked response is likely mediated through activating laryngeal Aδ‐fibers expressing MORs. However, IVb injection of FNT induces a more prolonged apnea coupled with hypotension and bradycardia, and importantly, this response occurs independently of the integrity of the SLN. Therefore, while activation of laryngeal MORs can induce a brief apnea, it is not the primary trigger of the apnea induced by IVb injection of FNT. These findings expand our current knowledge about the opioid‐induced respiratory disorder by revealing the distinct apneic responses elicited by FNT acting on laryngeal afferents' MORs. Furthermore, our data exclude the involvement of laryngeal afferents in the apneic response to IVb injection of FNT. Within vagal afferents, bronchopulmonary C‐fibers are also known to be capable of initiating apnea and vocal closure upon stimulation (Mutoh et al., 2000; Xu et al., 2023; Zhuang et al., 2012). This apnea can be greatly prolonged to a lethal extent during brief hypoxia (10% O_2_ for 1 min) (Xu et al., 2003). Thus, it is highly possible that bronchopulmonary C‐fibers are the key contributor to the long‐lasting apneic response to IVb injection of FNT. Confirmation of the contribution of bronchopulmonary C‐fibers to the prolonged apnea will provide valuable insights into the pathogenesis of sudden death associated with opioid overdose.

## CONFLICT OF INTEREST STATEMENT

All authors declare no conflict of interest.

## ETHICS STATEMENT

The experimental protocol (FY23‐010) was conducted in accordance with the Guide for the Care and Use of Laboratory Animals and approved by the Institutional Animal Care and Use Committee (IACUC), which is accredited by the Association for Assessment and Accreditation of Laboratory Animal Care International, USA.

## Data Availability

Data supporting this study are available on request to the authors.
